# Impact of body fat distribution on long-term clinical outcomes after drug-eluting stent implantation

**DOI:** 10.1371/journal.pone.0197991

**Published:** 2018-05-25

**Authors:** Se-Jun Park, Hong-Seok Lim, Seung-Soo Sheen, Hyoung-Mo Yang, Kyoung-Woo Seo, So-Yeon Choi, Byoung-Joo Choi, Myeong-Ho Yoon, Seung-Jea Tahk

**Affiliations:** 1 Division of Cardiology, Cardiovascular Center, Chun-Cheon Sacred Heart Hospital, Hallym University College of Medicine, Chun-Cheon, Republic of Korea; 2 Department of Cardiology, Ajou University School of Medicine, Suwon, Republic of Korea; 3 Department of Pulmonary and Critical Care Medicine, Ajou University School of Medicine, Suwon, Republic of Korea; CUNY, UNITED STATES

## Abstract

**Background and objective:**

The distribution of body fat is closely related to cardiovascular disease and outcomes, although its impact on patient prognosis after percutaneous coronary intervention (PCI) with drug-eluting stent (DES) has not been evaluated. We investigated the impact of truncal fat distribution on long-term clinical outcomes after DES treatment.

**Methods:**

In 441 DES-treated patients, dual energy X-ray absorptiometry was performed to assess total and regional body fat distribution after index PCI. The ratio of truncal fat to total body fat mass (%FM_trunk_/FM_total_) was calculated as a representative parameter for truncal fat distribution. The primary endpoint was major adverse cardiac events (MACE), a composite of ischemia-driven target vessel revascularization (TVR), non-procedural myocardial infarction, cardiac death at 5 years.

**Results:**

During the median follow-up duration of 1780 days, MACE occurred in 22.0% of patients, with the highest-quartile group of %FM_trunk_/FM_total_ having a higher rate than the lowest quartile group (27.8% vs. 15.3%; log rank *p* = 0.026). The difference was driven by a higher rate of ischemia-driven TVR (25.9% vs. 9.9%; log rank *p* = 0.008). In multivariable Cox regression analyses, %FM_trunk_/FM_total_ was independently associated with MACE (hazard ratio: 1.075; 95% CI: 1.022–1.131; *p* = 0.005), but body mass index (BMI) was not.

**Conclusions:**

In DES-treated patients, truncal fat distribution is associated with unfavorable clinical outcomes and is more clinically relevant than BMI.

## Introduction

Obesity is more than an increase in body weight; it is an important risk factor for the development of cardiovascular diseases (CVD). Furthermore, it is well known that as obesity increases, so does risk of mortality from CVD [1, 2]. Obesity is also strongly associated with the initiation and progression of atherosclerosis. In combination with the initiation and progression of *de novo* atherosclerosis, obesity may affect neoatherosclerosis after percutaneous coronary intervention (PCI) through various mechanisms, such as endothelial dysfunction and vascular inflammation. Thus, the obese population may have a worse prognosis than other populations [3]. In spite of the theoretical discussion of the harmful effects of obesity, the impact of obesity on clinical outcomes in patients who undergo PCI to treat coronary artery disease (CAD) has been debated. Many studies have suggested that obese patients have a better prognosis than their non-obese counterparts [4]. This “obesity paradox” has been reported in patients with various clinical conditions, particularly in those with CVD, the mechanism for which remains unclear [5]. Most likely this occurs as the result of selection bias, and so is not a true causal relation [6, 7]. Nevertheless, the prognostic role of adiposity remains an important question.

Central obesity is a more specific parameter to highlight the prognostic role of obesity on CVD than “simple” obesity; the latter is defined by body mass index (BMI). Furthermore, central fat distribution (i.e., visceral adipose tissue, VAT) has been directly linked to coronary atherothrombosis [8, 9] and therefore may be closely associated with the clinical outcomes of patients who undergo PCI. However, whether central fat distribution affects the prognosis of PCI-treated patients, specifically in those treated with a drug-eluting stent (DES), has not yet been evaluated.

We investigated the prognostic impact of central body fat distribution on long-term clinical outcomes in patients with CAD who underwent PCI with DES implantation.

## Methods

### Study population

From January 2005 to June 2008, we prospectively enrolled 441 consecutive patients who underwent both PCI with DES and dual X-ray absorptiometry (DXA). We excluded patients who underwent PCI with balloon angioplasty only or those who received a bare metal stent (S1 Fig). This study was approved by the Institutional Review Board of Ajou University Hospital, and all patients provided written informed consent.

### Assessment of body fat distribution

**Fig 1:**
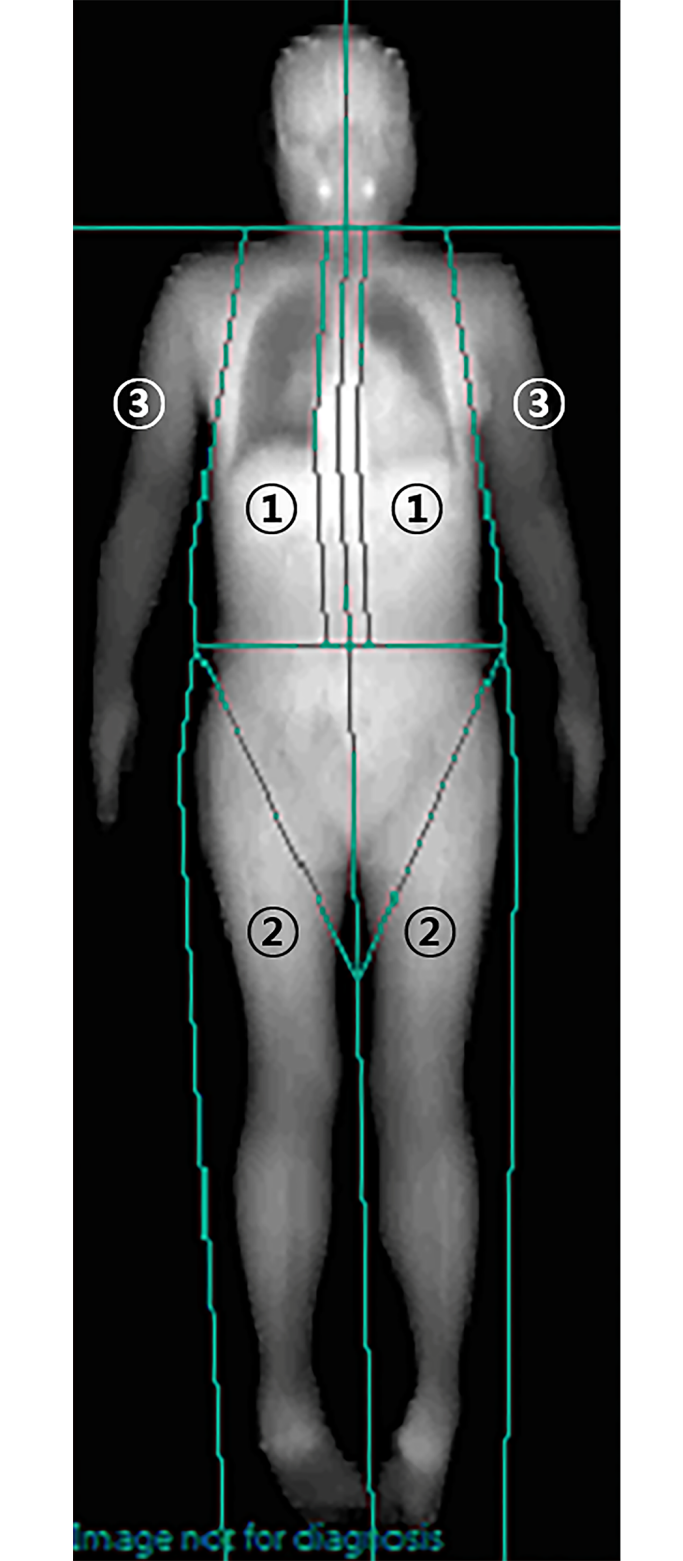
Regional fat measurement by dual-energy X-ray absorptiometry. 1: Trunk, the area bordered by the chin, the iliac crests, and the lateral borders of the ribs; 2: Legs, from the hip joints to the feet; 3: Arms, from lateral borders of the ribs to the arms.

The permeability of an X-ray is dependent on the thickness, density, and chemical composition of a tissue. DXA is used to estimate the mass of fat and lean tissues using high- and low-energy X-ray in body regions without bone [10]. Recent developments in software have enabled it to determine regional fat mass, which is comparable with computed tomography (CT), the gold standard imaging technique for the quantitative measurement of VAT [11]. One distinct advantage of DXA over CT is its ability to measure the relative distribution of body fat in a region of interest as it scans the whole body; indeed, DXA measures both total and regional body fat, and it does so with less radiation exposure to the patient [12]. Using DXA (Lunar Expert^™^, Madison, WI, USA), the body composition was obtained, as well as body fat mass, in specific areas (illustrated in Fig 1). The ratio of truncal fat mass to total body fat mass (%FM_trunk_/FM_total_) was calculated as a representative parameter for truncal fat distribution.

### Quantitative coronary angiography and percutaneous coronary intervention procedure

Coronary angiography was performed with standard techniques. Quantitative coronary angiographic analyses were performed in optimal projections using the Cardiovascular Angiography Analysis System II (Pie Medical Imaging, Maastricht, The Netherlands) by an experienced analyst who was unaware of the clinical status of the patients.

Before the procedure, aspirin and 300~600 mg of clopidogrel were administered to all patients. Heparin was administered during the procedure according to standard practice. All patients were recommended to maintain lifelong doses of aspirin (100mg/day) and clopidogrel (75mg/day) for at least 1 year after the index PCI. Patients received DES treatment using sirolimus-eluting stents (Cypher, Cordis Corporation, Johnson & Johnson, Warren, NJ, USA), paclitaxel-eluting stents (TAXUS Express or Liberté, Boston Scientific, Natick, MA, USA; Coroflex Please, B. Braun, Germany), everolimus-eluting stents (Promus Element, Boston Scientific; Xience Prime, Abbott Vascular, Santa Clara, CA, USA), and zotarolimus-eluting stents (Endeavor, Medtronic Vascular, Minneapolis, MN, USA).

### Patient follow-up and clinical outcome measurements

After the index PCI, all patients were followed-up with a visit to an outpatient clinic or by a telephone interview, when needed. The primary outcome was major adverse cardiac events (MACE) defined as composite of ischemia-driven target vessel revascularization (TVR), myocardial infarction (MI), and cardiac death. All clinical outcomes were defined according to the Academic Research Consortium and the third universal definition of MI. Ischemia-driven TVR was defined as any revascularization procedure of the target vessel prompted by symptoms or objective evidence of ischemia. MI was considered to be non-procedural myocardial damage, which was defined as an increase above the upper reference limit of creatine kinase-myocardial band or troponin level in the presence of relevant symptoms of acute coronary syndrome. Cardiac death was considered to be from a cardiac origin unless an undisputable non-cardiac cause was present.

### Statistical analysis

Categorical variables were described as their frequencies and percentages and continuous variables as their means and standard deviations or their medians and interquartile ranges. Student’s t test was used for the continuous variables, and chi-square test or Fisher’s exact test was used for the categorical variables. A Cox proportional hazard regression analyses were performed to identify independent predictors of MACE. Simple regression analyses using the primary endpoint as the response variable were performed for body fat indices and other known associated factors, which were categorized into either the demography and laboratory domain, coronary heart disease risk factor domain, medicine at discharge domain or angiographic and procedural factors domain. Covariates that showed a univariate relationship with *p* < 0.3 were entered into multiple regression analyses within each domain and over domains sequentially. The results were presented as a hazard ratio with 95% confidence intervals (CIs) and *p* value. Survival curves with cumulative events were prepared according to the Kaplan-Meier method and compared using the log-rank test. All analyses were performed using SPSS version 22.0 statistical software (IBM Corp, Armonk, NY, USA). A two-sided *p*-value < 0.05 was considered statistically significant.

## Results

### Baseline clinical characteristics and dual-energy X-ray absorptiometry findings

The baseline clinical and DXA findings are presented in Table 1. Among the 441 patients, the mean age was 60.0 ± 11.0 years, 63.0% were male, and 29.0% had diabetes mellitus (DM). Mean BMI was 25.0 ± 2.9 kg/m^2^ and ranged from 17.7 kg/m^2^ to 39.1 kg/m^2^. Total body fat mass and %FM_trunk_/FM_total_ of entire group of patients were 18.8 ± 6.3 kg and 59.6 ± 5.3%, respectively. The median BMI and %FM_trunk_/FM_total_ of the entire population were 24.8 kg/m^2^ and 60.3% with interquartile range of 23.1–26.9 and 56.3–63.4, respectively. Patients continued to take dual antiplatelet treatment for at least 12 months after DES implantation. Among all patients, 75.7%, 71.9% and 44.2% were taking a statin, renin-angiotensin system (RAS) blocker, and β-blocker at discharge, respectively.

**Table 1:** Baseline clinical characteristics and dual-energy X-ray absorptiometry measurements.

	Total (n = 441)	FM_trunk_/FM_total_			p value
		Low (n = 111)	Mid (n = 222)	High (n = 108)	
Clinical characteristics					
Age, years	60.0 ± 11.0	61.5 ± 12.0	60.8 ± 10.8	56.6 ± 9.4	0.001
Male, n (%)	278 (63.0%)	39 (35.1%)	143 (64.4%)	96 (88.9%)	< 0.001
DM, n (%)	128 (29.0%)	28 (25.2%)	57 (25.7%)	43 (39.8%)	0.018
Hypertension, n (%)	186 (42.2%)	52 (46.8%)	96 (43.2%)	38 (35.2%)	0.196
Dyslipidemia, n (%)	368 (83.4%)	84 (75.7%)	199 (89.6%)	85 (78.7%)	0.002
Current smoker, n (%)	144 (32.7%)	23 (20.7%)	73 (32.9%)	48 (44.4%)	0.001
Family history, n (%)	42 (9.5%)	12 (10.8%)	19 (8.6%)	11 (10.2%)	0.776
Body mass index, kg/m^2^	25.0 ± 2.9	23.7 ± 2.8	25.5 ± 2.9	25.3 ± 2.5	< 0.001
eGFR, mL/min/1.73m^2^	80.0 ± 21.7	80.5 ± 25.0	78.9 ± 20.2	81.5 ± 21.3	0.580
LVEF, %	63.8 ± 10.6	65.5 ± 10.8	63.5 ± 10.4	62.7 ± 10.8	0.130
Total cholesterol, mg/dL	170.6 ± 35.6	169.0 ± 40.7	173.4 ± 34.7	166.5 ± 31.2	0.225
Triglyceride, mg/dL	146.1 ± 120.8	118.0 ± 89.2	148.8 ± 137.8	169.3 ± 105.7	0.006
LDL-cholesterol, mg/dL	98.8 ± 28.7	98.5 ± 33.9	102.0 ± 26.6	92.8 ± 26.3	0.023
HDL-cholesterol, mg/dL	43.9 ± 10.6	46.8 ± 11.9	43.3 ± 10.2	42.0 ± 9.5	0.002
Clinical diagnosis					
STEMI, n (%)	41 (9.3%)	7 (6.3%)	16 (7.2%)	18 (16.7%)	0.010
NSTE-ACS, n (%)	305 (69.2%)	78 (70.3%)	156 (70.3%)	71 (65.7%)	0.676
Stable CAD, n (%)	95 (21.5%)	26 (23.4%)	50 (22.5%)	19 (17.6%)	0.508
Discharge medicine					
Statin, n (%)	334 (75.7%)	73 (65.8%)	170 (76.6%)	91 (84.3%)	0.006
RAS blocker, n (%)	317 (71.9%)	77 (69.4%)	161 (72.5%)	79 (73.1%)	0.788
β-blocker, n (%)	195 (44.2%)	50 (45.0%)	97 (43.7%)	48 (44.4%)	0.972
Dual energy X-ray absorptiometry					
Tissue mass, kg	62.9 ± 12.0	55.3 ± 9.5	64.4 ± 11.8	67.4 ± 11.2	< 0.001
Lean mass, kg	44.4 ± 9.5	38.5 ± 8.5	45.0 ± 9.1	49.2 ± 8.0	< 0.001
FM_total_, kg	18.8 ± 6.3	17.0 ± 6.8	19.7 ± 6.5	18.7 ± 4.6	0.001
FM_trunk_, kg	11.2 ± 3.9	8.9 ± 3.7	11.9 ± 3.9	12.3 ± 3.1	< 0.001
FM_arm_, kg	1.8 ± 0.8	1.9 ± 0.9	2.0 ± 0.8	1.6 ± 0.5	< 0.001
FM_leg_, kg	5.0 ± 1.9	5.4 ± 2.2	5.2 ± 1.8	4.1 ± 1.1	< 0.001
FM_trunk_/FM_total_, %	59.6 ± 5.3	52.3 ± 3.0	60.2 ± 2.0	65.8 ± 1.7	< 0.001

Values are expressed as mean ± standard deviation or number (%). DM, diabetes mellitus; eGFR, estimated glomerular filtration rate; LVEF, left ventricular ejection fraction; LDL, low density lipoprotein; HDL, high density lipoprotein; STEMI, ST-segment elevation myocardial infarction; NSTE-ACS, Non-ST-segment elevation acute coronary syndrome; CAD, coronary artery disease; RAS: renin-angiotensin system; FM_total_, total body fat mass; FM_trunk_, truncal fat mass; FM_arm_, fat mass in both arms; FM_leg_, fat mass in both legs.

With stratification by quartiles of %FM_trunk_/FM_total_ and BMI, we defined the three groups as follows: lowest quartile, low group; highest quartile, high group; and second and third quartiles, mid group. The high %FM_trunk_/FM_total_ group was younger and more likely to be diabetic and/or dyslipidemic with a higher rate of smokers compared with the other groups. Patients showed a decreased level of high-density lipoprotein and low-density lipoprotein cholesterols, but an increased level of triglycerides, along with increasing %FM_trunk_/FM_total_ values. Patients with a higher %FM_trunk_/FM_total_ were more likely to present with ST segment elevation myocardial infarction and to use statins at discharge.

### Angiographic lesion characteristics and percutaneous coronary intervention procedures

Coronary lesion characteristics and procedural findings are detailed in Table 2. In total, 917 DESs were implanted for 886 lesions; of these, 97.5% were first generation DESs. The mean stent diameter and total stent length per a patient were 3.2 ± 0.4 mm and 53.2 ± 35.2 mm, respectively. The angiographic and procedural characteristics did not differ significantly among the three groups.

**Table 2:** Coronary lesion characteristics and procedural findings.

	Total	FM_trunk_/FM_total_			p value
		Low	Mid	High	
Location of lesions					0.542
LM, n (%)	28 (3.2%)	4 (1.9%)	16 (3.5%)	8 (3.7%)	
LAD, n (%)	473 (53.4%)	126 (60.3%)	234 (51.0%)	113 (51.8%)	
LCX, n (%)	164 (18.5%)	34 (16.3%)	89 (19.4%)	41 (18.8%)	
RCA, n (%)	221 (24.9%)	45 (21.5%)	120 (26.1%)	56 (25.7%)	
Type of stent					0.377
SES, n (%)	623 (67.9%)	147 (67.1%)	317 (66.9%)	159 (71.0%)	
PES, n (%)	271 (29.6%)	68 (31.1%)	144 (30.4%)	59 (26.3%)	
ZES, n (%)	7 (0.8%)	1 (0.5%)	4 (0.8%)	2 (0.9%)	
EES, n (%)	16 (1.7%)	3 (1.4%)	9 (1.9%)	4 (1.8%)	
Type B2/C lesion, n^a^ (%)	357 (81.0%)	92 (82.9%)	177 (79.7%)	88 (81.5%)	0.778
Bifurcation, n^a^ (%)	92 (20.9%)	26 (23.4%)	48 (21.6%)	18 (16.7%)	0.434
Long lesion (≥20 mm), n^a^ (%)	275 (62.4%)	73 (65.8%)	142 (64.0%)	60 (55.6%)	0.232
CTO, n^a^ (%)	11 (2.5%)	5 (4.5%)	6 (2.7%)	0 (0.0%)	0.098
No. of diseased vessels					0.124
1-vessel disease, n (%)	257 (58.3%)	69 (62.2%)	128 (57.7%)	60 (55.6%)	
2-vessel disease, n (%)	139 (31.5%)	34 (30.6%)	64 (28.8%)	41 (38.0%)	
3-vessel disease, n (%)	45 (10.2%)	8 (7.2%)	30 (13.5%)	7 (6.5%)	
Multivessel disease, n (%)	184 (41.7%)	42 (37.8%)	94 (42.3%)	48 (44.4%)	0.591
Pre-PCI DS, %	81.4 ± 8.7	81.6 ± 7.8	80.9 ± 9.2	82.2 ± 8.7	0.404
Post-PCI DS, %	9.2 ± 5.1	8.9 ± 5.3	9.0 ± 4.9	10.0 ± 5.2	0.194
Reference diameter, mm	3.2 ± 0.4	3.2 ± 0.3	3.2 ± 0.4	3.3 ± 0.4	0.749
Total lesion length, mm	44.2 ± 29.0	43.2 ± 26.5	44.8 ± 30.3	43.9 ± 28.8	0.888
Average stent diameter, mm	3.2 ± 0.4	3.2 ± 0.3	3.2 ± 0.4	3.2 ± 0.4	0.489
Total stent length, mm^b^	53.2 ± 35.2	51.5 ± 31.9	54.0 ± 36.8	53.3 ± 35.5	0.838
No. of stents per patient, n	2.1 ± 1.2	2.0 ± 1.1	2.1 ± 1.3	2.1 ± 1.3	0.535

Values are expressed as mean ± standard deviation or number (%).

^a^Number of patients.

^b^Total stent length represents length of stents per a patient in total.

LM, left main; LAD, left anterior descending artery; LCX, left circumflex artery; RCA, right coronary artery; SES, sirolimus-eluting stent; PES, paclitaxel-eluting stent; ZES, zotarolimus-eluting stent; EES, everolimus-eluting stent; CTO, chronic total occlusion; PCI, percutaneous coronary intervention; MLD, minimal luminal diameter; DS, diameter stenosis.

### Clinical outcomes according to body fat distribution

The mean and median follow-up duration were 1884 ± 769 days and 1780 days, respectively. Clinical outcomes at 1, 3 and 5 years are described in Table 3.

**Table 3:** Cumulative rates of clinical events at 1, 3, and 5 years of follow-up.

	At 1 year	At 3 years	At 5 years
MACE, n (%)^a^	28 (6.3%)	71 (16.1%)	97 (22.0%)
TVR, n (%)	24 (5.4%)	57 (12.9%)	79 (17.9%)
MI, n (%)	1 (0.2%)	6 (1.4%)	7 (1.6%)
Cardiac death, n (%)	4 (0.9%)	19 (4.3%)	25 (5.7%)
Cardiac death or MI, n (%)	5 (1.1%)	25 (5.7%)	32 (7.3%)

Values are expressed as mean ± standard deviation and number (%).

^a^Major adverse cardiac events were defined as a composite of ischemia-driven target vessel revascularization, myocardial infarction and cardiac death.

MACE, major adverse cardiac event; TVR, ischemia-driven target vessel revascularization; MI, myocardial infarction.

**Fig 2:**
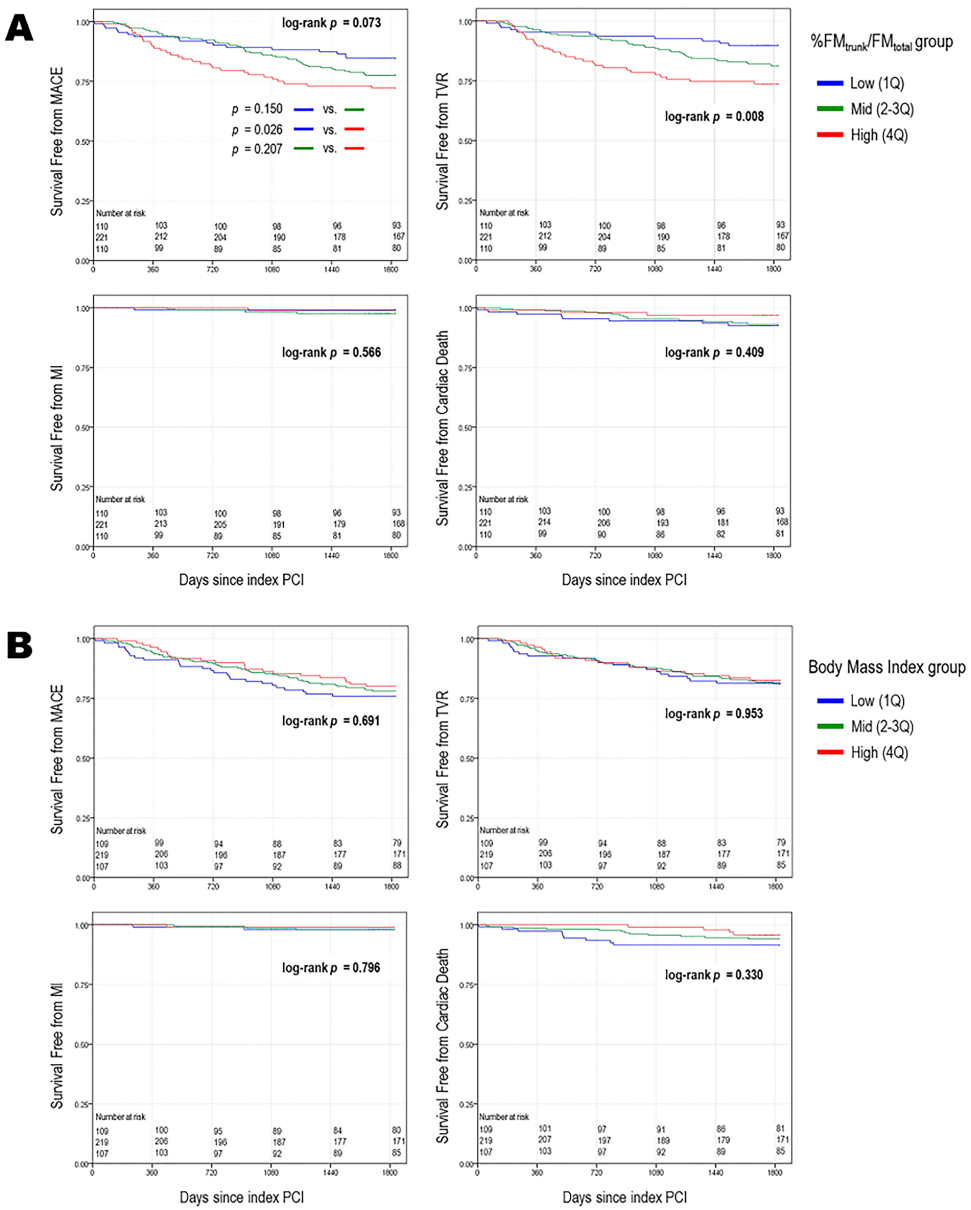
Kaplan–Meier curve for cumulative risk of event-free survival according to truncal fat distribution (A) and BMI (B). The cumulative survival rate of MACE, ischemia-driven TVR, MI, and cardiac death were compared among the low (first quartile), mid (second and third quartiles) and high (fourth quartile) groups stratified by %FM_trunk_/FM_total_ and BMI. BMI, body mass index; FM_total_, total body fat mass; FM_trunk_, fat mass in trunk; MACE, major adverse cardiac event; MI, myocardial infarction; TVR, target vessel revascularization.

According to Kaplan-Meier survival curves for the primary endpoint, there were no significant differences at 1 and 3 years among three groups of %FM_trunk_/FM_total_. However, the rate of MACE at 5 years tended to increase in groups with higher %FM_trunk_/FM_total_ (15.3% vs. 22.5% vs. 27.8%, log rank *p* = 0.073, Fig 2A). In post-hoc analysis, the low %FM_trunk_/FM_total_ group had a significantly lower 5-year MACE rate than the high group (log rank *p* = 0.026). Rates for individual components of the primary outcome were not different across the stratified groups of %FM_trunk_/FM_total_ except for that of ischemia-driven TVR among the high, mid, and low %FM_trunk_/FM_total_ groups (25.9%, 18.0%, and 9.9%; log rank *p* = 0.008, respectively).

By contrast, there were no significant differences in the occurrence rates of all of the endpoints throughout the duration of follow-up among the three groups categorized using the same method of stratification by quartiles of BMI (Fig 2B).

### Independent predictors of clinical outcomes

Simple and multiple regression analyses were performed to determine the factors independently associated with 5-year MACE (Table 4). According to the results of the multivariate model within domain, old age (≥ 65 years), sex, preserved left ventricular ejection fraction (≥ 50%), DM, hypertension, smoking, the use of statin, RAS blocker and β-blocker, long lesion (≥ 20 mm), FM_total_, and %FM_trunk_/FM_total_ remained within each domain and were included in the multivariable model over domain. The final multivariable Cox proportional hazards regression analyses showed that %FM_trunk_/FM_total_ was an independent predictor of MACE (hazard ratio: 1.075; 95% CI: 1.022–1.131; p = 0.005), along with old age (≥ 65 years), DM, hypertension, smoking, the use of statins and RAS blockers, and long lesion (≥ 20 mm). In the same analysis for individual endpoints, %FM_trunk_/FM_total_ was also identified as an adjusted predictor of ischemia-driven TVR (hazard ratio: 1.117; 95% CI: 1.055–1.183, p < 0.001). In contrast, BMI was not independently associated with either MACE or TVR.

**Table 4:** Univariable and multivariable Cox analyses to identify independent predictors of 5-year MACE.

Domain	Variable	Univariable model			Multivariable model within domain			Multivariable model over domain		
		HR	95% CI	p value	HR	95% CI	p value	HR	95% CI	p value
Demography and laboratory findings	Old age (≥ 65 years)	1.369	0.919–2.039	0.122	1.482	0.970–2.265	0.069	1.705	1.108–2.624	0.015
	Sex (male)	1.374	0.893–2.114	0.148	1.500	0.959–2.347	0.076	0.885	0.491–1.597	0.685
	BMI	0.990	0.925–1.060	0.777	–	–	–	–	–	–
	Total cholesterol	1.000	0.995–1.006	0.879	–	–	–	–	–	–
	Triglyceride	1.000	0.998–1.001	0.665	–	–	–	–	–	–
	LDL-cholesterol	1.001	0.994–1.008	0.842	–	–	–	–	–	–
	HDL-cholesterol	1.008	0.989–1.027	0.421	–	–	–	–	–	–
	eGFR	0.994	0.985–1.003	0.161	0.996	0.987–1.006	0.434	–	–	–
	Preserved LVEF (≥ 50%)	0.630	0.358–1.110	0.110	0.678	0.378–1.216	0.192	0.670	0.370–1.213	0.186
Risk factor for coronary heart disease	DM	1.740	1.160–2.612	0.007	1.693	1.121–2.555	0.012	1.595	1.038–2.449	0.033
	Hypertension	1.499	1.007–2.232	0.046	1.449	0.967–2.172	0.072	1.588	1.051–2.399	0.028
	Dyslipidemia	0.870	0.516–1.469	0.603	–	–	–	–	–	–
	Smoking	1.702	1.139–2.544	0.009	1.807	1.206–2.707	0.004	1.855	1.158–2.972	0.010
Medicine at discharge	Statin	0.687	0.447–1.057	0.088	0.724	0.469–1.117	0.145	0.602	0.382–0.949	0.029
	RAS blocker	0.678	0.447–1.029	0.068	0.708	0.465–1.078	0.107	0.586	0.378–0.909	0.017
	β-blocker	0.775	0.515–1.168	0.223	0.796	0.528–1.200	0.275	0.751	0.493–1.145	0.183
Angiographic and procedural findings	Multivessel disease	1.560	1.048–2.323	0.029	1.323	0.759–2.307	0.324	–	–	–
	Type B2/C lesion	1.113	0.659–1.878	0.690	–	–	–	–	–	–
	Long lesion (≥20 mm)	1.640	1.052–2.556	0.029	1.413	0.877–2.276	0.156	1.633	1.038–2.571	0.034
	Bifurcation	1.006	0.615–1.645	0.981	–	–	–	–	–	–
	Chronic total occlusion	0.757	0.187–3.073	0.697	–	–	–	–	–	–
	Mean reference diameter	0.669	0.388–1.151	0.146	0.789	0.440–1.415	0.427	–	–	–
	Average stent diameter	0.751	0.418–1.351	0.339	–	–	–	–	–	–
	No. of stents	1.165	1.008–1.346	0.038	1.022	0.821–1.271	0.848	–	–	–
Body fat parameters	FM_total_	0.979	0.947–1.012	0.203	0.973	0.939–1.008	0.125	0.980	0.944–1.018	0.309
	%FM_trunk_/FM_total_	1.053	1.010–1.097	0.015	1.055	1.013–1.100	0.010	1.075	1.022–1.131	0.005

MACE, major adverse cardiac events; HR, hazard ratio; CI, confidence interval; BMI, body mass index; LDL, low density lipoprotein; HDL, high density lipoprotein; eGFR, estimated glomerular filtration rate; LVEF, left ventricular ejection fraction; DM, diabetes mellitus; RAS, renin-angiotensin system; FM_total_, total fat mass; FM_trunk_, fat mass in trunk.

## Discussion

We investigated the prognostic impact of central fat distribution on long-term clinical outcomes in patients who underwent PCI with DES. Specifically, we observed that patients with higher truncal fat accumulation at the time of the index procedure have a more detrimental prognosis in MACE predominantly caused by ischemia-related TVR. To the best of our knowledge, this is the first study reporting the clinical relevance of body fat distribution on long-term clinical outcomes after DES implantation.

Obesity is strongly associated with the development, progression and subsequent clinical outcomes of atherosclerotic diseases [13]. Beyond metabolic derangement (i.e., insulin resistance, lipid dysregulation), obesity is accompanied by coronary atherosclerosis via inflammation and endothelial dysfunction [14]. Meanwhile, it has been proposed that vascular reaction after coronary stent implantation is more deleterious in obese patients, since vascular inflammation and dysregulated endothelium may have a pivot role in target vessel failure after PCI. Acute injuries by PCI results in vascular inflammation involving complex interactions with various cells that normally modulate vascular healing process. In pathological conditions, such as endothelial injury by stenting, a dysregulated vascular response induces neointimal hyperplasia. Accordingly, these reactions are prompted by endothelial dysfunction and hypercoagulable status derived from persistent inflammation [3, 15].

PCI via coronary stenting is now an indispensable for the treatment of CAD. Since coronary stents were first introduced to overcome the shortcomings of balloon angioplasty by maintaining luminal integrity, DES was developed and briskly adopted into clinical practice. Indeed, DES exhibits a significant reduction in neointimal proliferation and in-stent restenosis in various clinical indications [16]. Even in the current era of DES ushering more patients to be better candidates for PCI with ensuring low rates of subsequent revascularization procedures; however, despite this, late complications remain a concern, and much work has been done to further reduce the rate of late stent failure. By improving the stent platform, second-generation DES has had better performances for PCI compared to first-generation DES, which led to favorable clinical outcomes [17]. The coated drug and polymer might induce incomplete re-endothelization and chronic inflammatory reaction, which occasionally lead to stent thrombosis or neoatherosclerosis and increased risks of late clinical event [3]. Specifically, neoatherosclerosis is considered an important mechanism of late DES failure, whose initial changes may affect long-term outcomes. An optical-coherence tomography study showed that in-stent neoatherosclerosis, including lipid-rich neointima, is developed earlier in DES than in bare metal stent [18], whereas the autopsy study reported no difference of that between first- and second-generation DES [19]. Central obesity has been recognized as more relevant to the prognosis of CVD than BMI. Of the 6,560 patients in the MERLIN-TIMI-36 study, those with waist circumference (WCs) greater than proportionate to BMI were at the highest risk of developing cardiovascular events [20]. In addition, a systemic review of 15,923 patients with CAD showed that WC or waist-to-hip ratio is associated with mortality, unlike BMI [21]. As a major component of central obesity, abdominal visceral fat promotes atherogenesis and has harmful effects on CVD progression, such as vulnerable coronary plaque that causes subsequent MI [22]. Epicardial adipose tissue (EAT) is another offender, which has a greater local effect on both the quality and quantity of coronary atherosclerosis [23]. Moreover, higher EAT increases the risk of target lesion revascularization after PCI [24]. Body fat accumulated in the trunk which reflects abdominal VAT and EAT together, also have significant association with the extent of coronary atherosclerosis [9]. Truncal fat has two distinct compartments: VAT and subcutaneous fat, which is further divided into superficial and deep subcutaneous fat. In contrast with VAT, subcutaneous fat is generally protective for cardiometabolic complications; however, deep subcutaneous fat (not superficial) has similar properties to VAT [23].

The present study demonstrated that the relative truncal fat distribution representing the integrated amount of VAT and EAT is a more important predictor of prognosis than the absolute amount of total body fat or BMI in DES-treated patients. This is a novel finding with clinical significance. The detrimental effects of truncal fat were mainly appeared as repeat revascularization and became obvious during long-term follow-up rather than < 1 year after index PCI.

Notably, few studies to date have examined the overall impact of obesity or fat distribution on patient outcomes after DES implantations, particularly over long-term follow-up. Given this context, this study is even more useful. Although the long-term predictive value of %FM_trunk_/FM_total_ is not satisfactory enough for it to be solely relied upon in clinical practice, the mean %FM_trunk_/FM_total_ of the patients in the highest quartile, interestingly, was 65.8%, which was similar to the criteria for predicting 5-year MACE (66.1%). This study was not without limitations. First, the distribution of body fat at a single point of time might not reflect long-term changes in it. However, central obesity or body composition cannot easily be improved, because obesity-associated behavioral changes are induced by a greater-than-normal weight gain and newly developed obesity-related morbidities [25]. Moreover, because body fat distribution at the time of the index procedure influences the recovery of endothelial injury immediately after PCI, it might be of greater importance in the overall healing process than in the later period of follow-up. Because of the cross-sectional assessment of the patients’ baseline features, our study could not provide information regarding the association between changes in patients’ lifestyle or physical fitness and clinical outcomes. Second, DXA is not yet a gold standard method for measuring body fat distribution. However, DXA can detect the whole-body distribution of various compositions in less time and with less radiation and expense than CT [12]. Unlike CT, DXA is unable to discriminate subcutaneous fat from VAT; however, in regard to VAT, the discrepancy tends to be lesser as the VAT volume increases [26].

Given its verified reliability, which is comparable with that of CT, DXA has been used recently in many studies on body fat distribution [27]. Third, the first-generation DESs were used in almost all patients. Thus, our results are not generalizable to the current, newer generation DES era. Despite the improved safety and efficacy of the second-generation DESs, there was no difference of long-term clinical outcomes between two DES types [28]. Further studies are needed.

## Conclusion

Body fat distribution is associated with clinical outcomes of patients who underwent PCI with DES. Truncal fat accumulation, which reflects visceral and epicardial adiposity en bloc, is an independent predictor of long-term cardiovascular prognosis in those populations, but BMI is not.

## Supporting information

S1 Fig Study enrollment and exclusion. (TIF)

S1 File De-identified data set. (XLSX)
