# Supplementary figures and images for: Impact of body fat distribution on long-term clinical outcomes after drug-eluting stent implantation
Source: PLoS One. 2018 May 25;13(5):e0197991. doi: 10.1371/journal.pone.0197991 (PMC5969753; doi:10.1371/journal.pone.0197991)

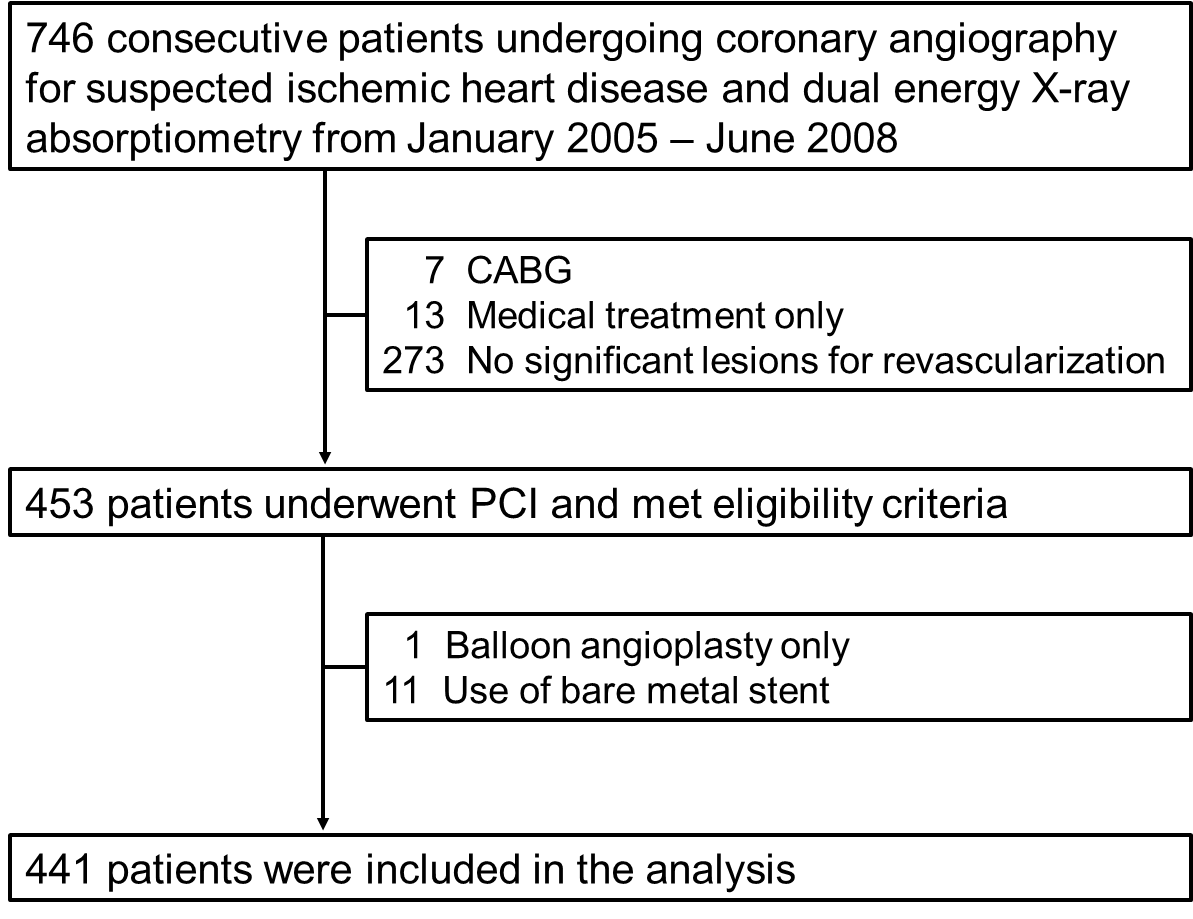

Supplement: S1 Fig — (TIF) [file pone.0197991.s001.tif]
